# (*E*)-*N*′-(4-Eth­oxy­benzyl­idene)-4-hy­droxy­benzohydrazide dihydrate

**DOI:** 10.1107/S1600536812019356

**Published:** 2012-05-05

**Authors:** Hoong-Kun Fun, Jirapa Horkaew, Suchada Chantrapromma, Chatchanok Karalai

**Affiliations:** a X-ray Crystallography Unit, School of Physics, Universiti Sains Malaysia, 11800 USM, Penang, Malaysia; b Crystal Materials Research Unit, Department of Chemistry, Faculty of Science, Prince of Songkla University, Hat-Yai, Songkhla 90112, Thailand

## Abstract

The benzohydrazide mol­ecule of the title compound, C_16_H_16_N_2_O_3_·2H_2_O, exists in a *trans* conformation with respect to the C=N double bond. The central O=C—NH—N=C plane [r.m.s. deviation of 0.0165 (1) Å for the five non-H atoms] makes dihedral angles of 6.04 (8) and 2.38 (8)°, respectively, with the hy­droxy- and eth­oxy-substituted benzene rings. The dihedral angle between these benzene rings is 3.82 (7)°. The eth­oxy group is almost coplanar with the attached benzene ring with a C—O—C—C torsion angle of −176.58 (11)°. In the crystal, the benzohydrazide and water mol­ecules are linked by N—H⋯O, O—H⋯O , O—H⋯N and C—H⋯O hydrogen bonds into a three-dimensional network.

## Related literature

For bond-length data, see: Allen *et al.* (1987 ▶). For related structures, see: Fun *et al.* (2011 ▶); Horkaew *et al.* (2011 ▶, 2012 ▶). For applications of benzohydrazides, see: Loncle *et al.* (2004 ▶); Molyneux (2004 ▶); Promdet *et al.* (2011 ▶); Raj *et al.* (2007 ▶). For the stability of the temperature controller used in the data collection, see: Cosier & Glazer (1986 ▶).
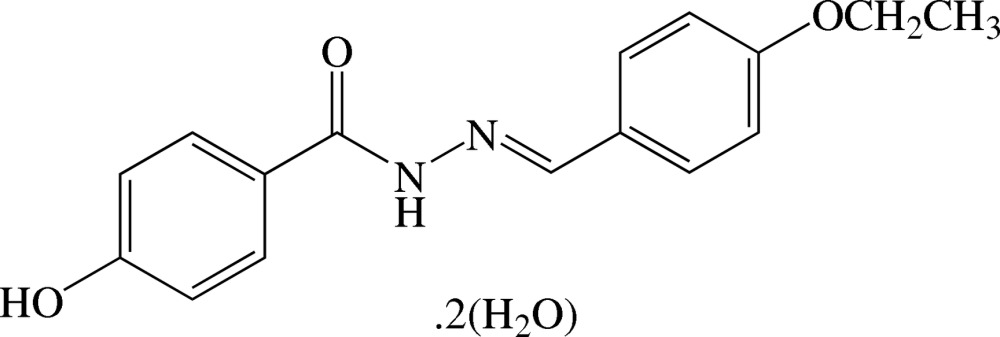


## Experimental

### 

#### Crystal data

C_16_H_16_N_2_O_3_·2H_2_O
*M* *_r_* = 320.34
Monoclinic, 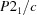

*a* = 7.1655 (1) Å
*b* = 17.3895 (3) Å
*c* = 13.6202 (2) Å
β = 110.875 (1)°
*V* = 1585.74 (4) Å^3^
*Z* = 4
Mo *K*α radiation
μ = 0.10 mm^−1^
*T* = 100 K
0.29 × 0.16 × 0.16 mm

#### Data collection

Bruker APEXII CCD area-detector diffractometer
Absorption correction: multi-scan (*SADABS*; Bruker, 2005 ▶) *T*_min_ = 0.971, *T*_max_ = 0.984
22228 measured reflections
5737 independent reflections
4310 reflections with *I* > 2σ(*I*)
*R*_int_ = 0.032

#### Refinement

*R*[*F* ^2^ > 2σ(*F* ^2^)] = 0.055
*wR*(*F* ^2^) = 0.155
*S* = 1.03
5737 reflections
209 parameters
H-atom parameters constrained
Δρ_max_ = 0.73 e Å^−3^
Δρ_min_ = −0.28 e Å^−3^

### 

Data collection: *APEX2* (Bruker, 2005 ▶); cell refinement: *SAINT* (Bruker, 2005 ▶); data reduction: *SAINT*; program(s) used to solve structure: *SHELXTL* (Sheldrick, 2008 ▶); program(s) used to refine structure: *SHELXTL*; molecular graphics: *SHELXTL*; software used to prepare material for publication: *SHELXTL* and *PLATON* (Spek, 2009 ▶).

## Supplementary Material

Crystal structure: contains datablock(s) global, I. DOI: 10.1107/S1600536812019356/is5130sup1.cif

Structure factors: contains datablock(s) I. DOI: 10.1107/S1600536812019356/is5130Isup2.hkl

Supplementary material file. DOI: 10.1107/S1600536812019356/is5130Isup3.cml

Additional supplementary materials:  crystallographic information; 3D view; checkCIF report

## Figures and Tables

**Table 1:** Hydrogen-bond geometry (Å, °)

*D*—H⋯*A*	*D*—H	H⋯*A*	*D*⋯*A*	*D*—H⋯*A*
O2—H1⋯O1^i^	0.84	1.77	2.6106 (15)	174
N1—H2⋯O1*W*^ii^	0.85	2.10	2.9278 (16)	167
O1*W*—H3⋯O2^iii^	0.81	2.06	2.8683 (15)	177
O1*W*—H4⋯O2*W*	0.88	1.84	2.7159 (19)	169
O2*W*—H5⋯O1^iv^	0.87	2.14	2.8558 (18)	139
O2*W*—H5⋯N2^iv^	0.87	2.55	3.3363 (19)	151
O2*W*—H6⋯O3^v^	0.89	2.11	2.9791 (17)	165
C6—H6*A*⋯O1*W*^ii^	0.95	2.36	3.2942 (18)	169
C8—H8*A*⋯O1*W*^ii^	0.95	2.49	3.3222 (18)	146

Symmetry codes: (i) 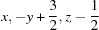; (ii) 
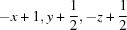
; (iii) 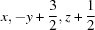; (iv) 
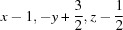
; (v) 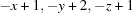.

**Fig. 1.**
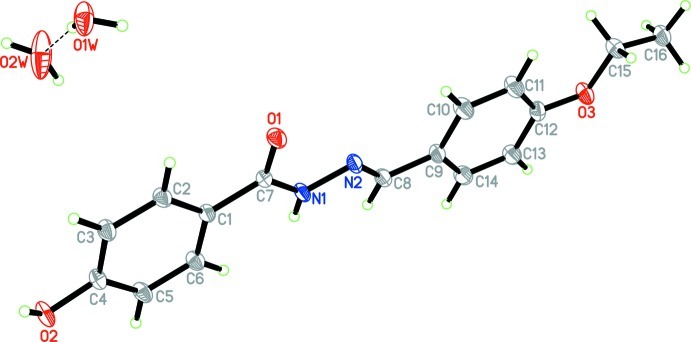
The molecular structure of the title compound, showing 60% probability displacement ellipsoids and the atom-numbering scheme. The O—H···O hydrogen bond is shown as a dashed line.

**Fig. 2.**
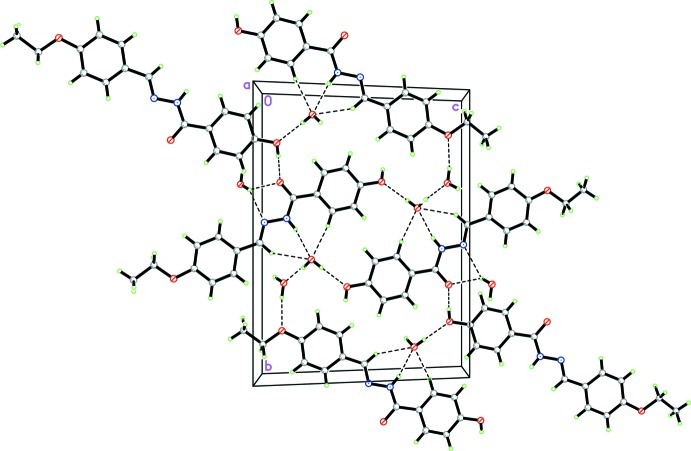
The crystal packing of the title compound viewed along the a axis. Hydrogen bonds are shown as dashed lines.

## supplementary crystallographic information

### Comment

It has been known that a majority of benzohydrazides possesses various biological properties, such as antibacterial and antifungal (Loncle *et al.*, 2004), and antiproliferative (Raj *et al.*, 2007) activities. The title benzohydrazide derivative (I) was synthesized as part of our study on the bioactivity of benzohydrazide derivatives (Fun *et al.*, 2011; Horkaew *et al.*, 2011, 2012; Promdet *et al.*, 2011) and was evaluated for antioxidant activity by DPPH scavenging (Molyneux, 2004). It was found to be active. Herein we report the synthesis and crystal structure of (I).

The title compound (Fig. 1), C_16_H_16_N_2_O_3_.2H_2_O, comprises one benzohydrazide molecule and two water molecules. The molecule of benzohydrazide exists in a *trans*-configuration with respect to the C8═N2 bond and the torsion angle N1—N2—C8—C9 = -179.90 (11)° with the dihedral angle between the two benzene rings being 3.82 (7)°. The middle fragment are planar with an *r.m.s*. deviation of 0.0165 (1) Å for the five non-H atoms (O1, C7, N1, N2 and C8). The mean plane through this middle fragment makes the dihedral angles of 6.04 (8) and 2.38 (8)° with the 4-hydroxyphenyl and 4-ethoxyphenyl rings, respectively. The ethoxy group is co-planar with the bound benzene ring with the torsion angle C12—O3—C15—C16 = -176.58 (11)°. The molecule is therefore approximately planar. The two water molecules are linked to each other by an O—H···O hydrogen bond (Fig. 1). Bond distances of benzohydrazide are of normal values (Allen *et al.*, 1987) and are comparable with the related structures (Fun *et al.*, 2011; Horkaew *et al.*, 2011, 2012).

In the crystal packing (Fig. 2), the molecules of benzohydrazide and water are linked by N—H···O, O—H···O, O—H···N and C—H···O hydrogen bonds (Table 1) into a three-dimensional network.

### Experimental

The title compound (I) was prepared by dissolving 4-hydroxybenzohydrazide (2 mmol, 0.30 g) in ethanol (15 ml). A solution of 4-ethoxybenzaldehyde (2 mmol, 0.27 ml) in ethanol (15 ml) was then added slowly to the reaction. The mixture was refluxed for around 6 hr. The solution was then cooled to room temperature and a white solid appeared. Colorless block-shaped single crystals of the title compound suitable for *X*-ray structure determination were recrystallized from a methanol solution by slow evaporation of the solvent at room temperature after several days (m.p. 373-374 K).

### Refinement

All H atoms were positioned geometrically [*d*(O—H) = 0.84 Å for the hydroxy group and 0.81–0.89 Å for water molecules, *d*(N—H) = 0.85 Å, *d*(C—H) = 0.95 Å for aromatic and CH, 0.99 Å for CH_2_ and 0.98 Å for CH_3_ groups] and allowed to ride on their parent atoms, The *U*_iso_(H) values were constrained to be 1.5*U*_eq_ of the carrier atom for methyl H atoms and 1.2*U*_eq_ for the remaining H atoms. A rotating group model was used for the methyl group.

### Crystal data

**Table:**

C_16_H_16_N_2_O_3_·2H_2_O	*F*(000) = 680
*M**_r_* = 320.34	*D*_x_ = 1.342 Mg m^−^^3^
Monoclinic, *P*2_1_/*c*	Melting point = 373–374 K
Hall symbol: -P 2ybc	Mo *K*α radiation, λ = 0.71073 Å
*a* = 7.1655 (1) Å	Cell parameters from 5737 reflections
*b* = 17.3895 (3) Å	θ = 2.0–32.5°
*c* = 13.6202 (2) Å	µ = 0.10 mm^−^^1^
β = 110.875 (1)°	*T* = 100 K
*V* = 1585.74 (4) Å^3^	Block, colorless
*Z* = 4	0.29 × 0.16 × 0.16 mm

### Data collection

**Table:**

Bruker APEXII CCD area-detector diffractometer	5737 independent reflections
Radiation source: sealed tube	4310 reflections with *I* > 2σ(*I*)
Graphite monochromator	*R*_int_ = 0.032
φ and ω scans	θ_max_ = 32.5°, θ_min_ = 2.0°
Absorption correction: multi-scan (*SADABS*; Bruker, 2005)	*h* = −10→10
*T*_min_ = 0.971, *T*_max_ = 0.984	*k* = −26→19
22228 measured reflections	*l* = −20→18

### Refinement

**Table:**

Refinement on *F*^2^	Primary atom site location: structure-invariant direct methods
Least-squares matrix: full	Secondary atom site location: difference Fourier map
*R*[*F*^2^ > 2σ(*F*^2^)] = 0.055	Hydrogen site location: inferred from neighbouring sites
*wR*(*F*^2^) = 0.155	H-atom parameters constrained
*S* = 1.03	*w* = 1/[σ^2^(*F*_o_^2^) + (0.0735*P*)^2^ + 0.7894*P*] where *P* = (*F*_o_^2^ + 2*F*_c_^2^)/3
5737 reflections	(Δ/σ)_max_ = 0.001
209 parameters	Δρ_max_ = 0.73 e Å^−^^3^
0 restraints	Δρ_min_ = −0.28 e Å^−^^3^

### Special details

**Table:**

Experimental. The crystal was placed in the cold stream of an Oxford Cryosystems Cobra open-flow nitrogen cryostat (Cosier & Glazer, 1986) operating at 120.0 (1) K.
Geometry. All esds (except the esd in the dihedral angle between two l.s. planes) are estimated using the full covariance matrix. The cell esds are taken into account individually in the estimation of esds in distances, angles and torsion angles; correlations between esds in cell parameters are only used when they are defined by crystal symmetry. An approximate (isotropic) treatment of cell esds is used for estimating esds involving l.s. planes.
Refinement. Refinement of F^2^ against ALL reflections. The weighted R-factor wR and goodness of fit S are based on F^2^, conventional R-factors R are based on F, with F set to zero for negative F^2^. The threshold expression of F^2^ > 2sigma(F^2^) is used only for calculating R-factors(gt) etc. and is not relevant to the choice of reflections for refinement. R-factors based on F^2^ are statistically about twice as large as those based on F, and R- factors based on ALL data will be even larger.

### Fractional atomic coordinates and isotropic or equivalent isotropic displacement parameters (Å^2^)

**Table:**

	*x*	*y*	*z*	*U*_iso_*/*U*_eq_	
O1	0.75546 (15)	0.81977 (6)	0.40609 (8)	0.0174 (2)	
O2	0.60182 (15)	0.81464 (6)	−0.08291 (8)	0.0188 (2)	
H1	0.6435	0.7704	−0.0894	0.028*	
O3	0.85596 (15)	1.15548 (6)	0.90045 (8)	0.0198 (2)	
N1	0.70782 (16)	0.94655 (7)	0.37373 (9)	0.0146 (2)	
H2	0.6729	0.9842	0.3315	0.018*	
N2	0.75068 (16)	0.96088 (7)	0.47938 (9)	0.0151 (2)	
C1	0.67580 (17)	0.86049 (7)	0.22842 (10)	0.0130 (2)	
C2	0.67471 (19)	0.78444 (8)	0.19463 (11)	0.0156 (2)	
H2A	0.6925	0.7437	0.2436	0.019*	
C3	0.64817 (19)	0.76747 (8)	0.09099 (11)	0.0162 (2)	
H3A	0.6461	0.7155	0.0690	0.019*	
C4	0.62471 (18)	0.82718 (8)	0.01968 (10)	0.0145 (2)	
C5	0.62192 (19)	0.90311 (8)	0.05149 (11)	0.0163 (2)	
H5A	0.6018	0.9436	0.0020	0.020*	
C6	0.64847 (19)	0.91980 (8)	0.15534 (11)	0.0156 (2)	
H6A	0.6481	0.9718	0.1768	0.019*	
C7	0.71456 (17)	0.87382 (7)	0.34201 (10)	0.0127 (2)	
C8	0.73020 (19)	1.03161 (8)	0.50069 (11)	0.0162 (2)	
H8A	0.6885	1.0676	0.4446	0.019*	
C9	0.76804 (19)	1.05918 (8)	0.60712 (11)	0.0155 (2)	
C10	0.8108 (2)	1.01088 (8)	0.69423 (11)	0.0200 (3)	
H10A	0.8197	0.9569	0.6858	0.024*	
C11	0.8404 (2)	1.04100 (8)	0.79300 (11)	0.0201 (3)	
H11A	0.8681	1.0077	0.8517	0.024*	
C12	0.82933 (18)	1.12032 (8)	0.80608 (11)	0.0166 (2)	
C13	0.7880 (2)	1.16903 (8)	0.72028 (11)	0.0186 (3)	
H13A	0.7809	1.2230	0.7291	0.022*	
C14	0.7570 (2)	1.13863 (8)	0.62170 (11)	0.0189 (3)	
H14A	0.7279	1.1721	0.5631	0.023*	
C15	0.9043 (2)	1.10800 (9)	0.99250 (11)	0.0196 (3)	
H15A	1.0348	1.0825	1.0066	0.024*	
H15B	0.8012	1.0678	0.9820	0.024*	
C16	0.9131 (2)	1.15890 (9)	1.08336 (11)	0.0207 (3)	
H16A	0.9521	1.1283	1.1479	0.031*	
H16B	0.7815	1.1818	1.0702	0.031*	
H16C	1.0116	1.1998	1.0912	0.031*	
O1W	0.36756 (19)	0.59100 (6)	0.24443 (9)	0.0301 (3)	
H3	0.4370	0.6177	0.2921	0.045*	
H4	0.2664	0.6197	0.2059	0.045*	
O2W	0.0731 (2)	0.67607 (8)	0.10548 (11)	0.0471 (4)	
H5	−0.0331	0.6558	0.0590	0.071*	
H6	0.0705	0.7267	0.0963	0.071*	

### Atomic displacement parameters (Å^2^)

**Table:**

	*U*^11^	*U*^22^	*U*^33^	*U*^12^	*U*^13^	*U*^23^
O1	0.0255 (5)	0.0134 (5)	0.0125 (4)	−0.0004 (3)	0.0059 (4)	0.0019 (4)
O2	0.0281 (5)	0.0179 (5)	0.0100 (4)	0.0064 (4)	0.0062 (4)	−0.0004 (4)
O3	0.0277 (5)	0.0181 (5)	0.0126 (5)	0.0025 (4)	0.0059 (4)	−0.0014 (4)
N1	0.0212 (5)	0.0134 (5)	0.0086 (5)	0.0002 (4)	0.0047 (4)	0.0005 (4)
N2	0.0184 (5)	0.0170 (5)	0.0097 (5)	−0.0007 (4)	0.0044 (4)	−0.0015 (4)
C1	0.0142 (5)	0.0133 (6)	0.0111 (5)	0.0001 (4)	0.0041 (4)	−0.0003 (4)
C2	0.0220 (6)	0.0125 (6)	0.0129 (6)	0.0000 (4)	0.0067 (5)	0.0008 (5)
C3	0.0223 (6)	0.0132 (6)	0.0132 (6)	0.0008 (4)	0.0063 (5)	−0.0010 (5)
C4	0.0159 (5)	0.0165 (6)	0.0103 (6)	0.0025 (4)	0.0037 (4)	0.0000 (4)
C5	0.0227 (6)	0.0142 (6)	0.0123 (6)	0.0039 (4)	0.0067 (5)	0.0025 (5)
C6	0.0211 (5)	0.0129 (6)	0.0134 (6)	0.0024 (4)	0.0070 (5)	0.0012 (5)
C7	0.0137 (5)	0.0139 (6)	0.0105 (5)	−0.0006 (4)	0.0042 (4)	0.0000 (4)
C8	0.0203 (5)	0.0161 (6)	0.0125 (6)	−0.0015 (4)	0.0061 (5)	−0.0004 (5)
C9	0.0173 (5)	0.0176 (6)	0.0117 (6)	−0.0005 (4)	0.0052 (4)	−0.0018 (5)
C10	0.0275 (6)	0.0163 (6)	0.0162 (7)	0.0028 (5)	0.0078 (5)	0.0004 (5)
C11	0.0274 (6)	0.0175 (6)	0.0140 (6)	0.0030 (5)	0.0055 (5)	0.0020 (5)
C12	0.0166 (5)	0.0184 (6)	0.0139 (6)	−0.0005 (4)	0.0043 (5)	−0.0032 (5)
C13	0.0246 (6)	0.0145 (6)	0.0168 (7)	0.0002 (5)	0.0075 (5)	−0.0005 (5)
C14	0.0247 (6)	0.0154 (6)	0.0169 (6)	−0.0012 (5)	0.0080 (5)	−0.0005 (5)
C15	0.0236 (6)	0.0212 (7)	0.0144 (6)	0.0014 (5)	0.0073 (5)	0.0014 (5)
C16	0.0235 (6)	0.0247 (7)	0.0137 (6)	−0.0009 (5)	0.0066 (5)	−0.0027 (5)
O1W	0.0463 (7)	0.0148 (5)	0.0187 (6)	0.0027 (5)	−0.0014 (5)	−0.0031 (4)
O2W	0.0469 (8)	0.0341 (8)	0.0342 (8)	0.0181 (6)	−0.0176 (6)	−0.0151 (6)

### Geometric parameters (Å, º)

**Table:**

O1—C7	1.2445 (16)	C8—H8A	0.9500
O2—C4	1.3651 (16)	C9—C10	1.3957 (19)
O2—H1	0.8415	C9—C14	1.402 (2)
O3—C12	1.3738 (16)	C10—C11	1.388 (2)
O3—C15	1.4368 (17)	C10—H10A	0.9500
N1—C7	1.3428 (17)	C11—C12	1.397 (2)
N1—N2	1.3828 (15)	C11—H11A	0.9500
N1—H2	0.8479	C12—C13	1.387 (2)
N2—C8	1.2841 (18)	C13—C14	1.385 (2)
C1—C6	1.3977 (18)	C13—H13A	0.9500
C1—C2	1.3994 (18)	C14—H14A	0.9500
C1—C7	1.4894 (18)	C15—C16	1.504 (2)
C2—C3	1.3872 (19)	C15—H15A	0.9900
C2—H2A	0.9500	C15—H15B	0.9900
C3—C4	1.3906 (19)	C16—H16A	0.9800
C3—H3A	0.9500	C16—H16B	0.9800
C4—C5	1.3920 (19)	C16—H16C	0.9800
C5—C6	1.3890 (19)	O1W—H3	0.8092
C5—H5A	0.9500	O1W—H4	0.8829
C6—H6A	0.9500	O2W—H5	0.8715
C8—C9	1.4575 (18)	O2W—H6	0.8889
			
C4—O2—H1	109.6	C10—C9—C8	123.66 (13)
C12—O3—C15	118.06 (11)	C14—C9—C8	117.71 (12)
C7—N1—N2	118.98 (11)	C11—C10—C9	120.55 (13)
C7—N1—H2	123.0	C11—C10—H10A	119.7
N2—N1—H2	117.9	C9—C10—H10A	119.7
C8—N2—N1	114.02 (12)	C10—C11—C12	120.03 (13)
C6—C1—C2	118.69 (12)	C10—C11—H11A	120.0
C6—C1—C7	123.50 (12)	C12—C11—H11A	120.0
C2—C1—C7	117.77 (11)	O3—C12—C13	115.67 (12)
C3—C2—C1	121.23 (12)	O3—C12—C11	124.31 (13)
C3—C2—H2A	119.4	C13—C12—C11	120.02 (13)
C1—C2—H2A	119.4	C14—C13—C12	119.73 (13)
C2—C3—C4	119.37 (12)	C14—C13—H13A	120.1
C2—C3—H3A	120.3	C12—C13—H13A	120.1
C4—C3—H3A	120.3	C13—C14—C9	121.04 (13)
O2—C4—C3	122.42 (12)	C13—C14—H14A	119.5
O2—C4—C5	117.41 (12)	C9—C14—H14A	119.5
C3—C4—C5	120.16 (12)	O3—C15—C16	107.82 (12)
C6—C5—C4	120.21 (12)	O3—C15—H15A	110.1
C6—C5—H5A	119.9	C16—C15—H15A	110.1
C4—C5—H5A	119.9	O3—C15—H15B	110.1
C5—C6—C1	120.31 (12)	C16—C15—H15B	110.1
C5—C6—H6A	119.8	H15A—C15—H15B	108.5
C1—C6—H6A	119.8	C15—C16—H16A	109.5
O1—C7—N1	120.81 (12)	C15—C16—H16B	109.5
O1—C7—C1	121.40 (12)	H16A—C16—H16B	109.5
N1—C7—C1	117.78 (11)	C15—C16—H16C	109.5
N2—C8—C9	122.96 (13)	H16A—C16—H16C	109.5
N2—C8—H8A	118.5	H16B—C16—H16C	109.5
C9—C8—H8A	118.5	H3—O1W—H4	106.9
C10—C9—C14	118.62 (12)	H5—O2W—H6	109.2
			
C7—N1—N2—C8	−176.66 (11)	N1—N2—C8—C9	−179.90 (11)
C6—C1—C2—C3	0.32 (18)	N2—C8—C9—C10	−6.5 (2)
C7—C1—C2—C3	−177.23 (11)	N2—C8—C9—C14	174.70 (12)
C1—C2—C3—C4	0.81 (19)	C14—C9—C10—C11	0.4 (2)
C2—C3—C4—O2	178.72 (11)	C8—C9—C10—C11	−178.38 (12)
C2—C3—C4—C5	−1.92 (19)	C9—C10—C11—C12	−0.6 (2)
O2—C4—C5—C6	−178.70 (11)	C15—O3—C12—C13	−178.41 (11)
C3—C4—C5—C6	1.91 (19)	C15—O3—C12—C11	1.97 (18)
C4—C5—C6—C1	−0.77 (19)	C10—C11—C12—O3	179.87 (12)
C2—C1—C6—C5	−0.34 (18)	C10—C11—C12—C13	0.3 (2)
C7—C1—C6—C5	177.06 (11)	O3—C12—C13—C14	−179.40 (12)
N2—N1—C7—O1	1.17 (17)	C11—C12—C13—C14	0.2 (2)
N2—N1—C7—C1	−177.57 (10)	C12—C13—C14—C9	−0.4 (2)
C6—C1—C7—O1	−173.58 (12)	C10—C9—C14—C13	0.1 (2)
C2—C1—C7—O1	3.85 (17)	C8—C9—C14—C13	178.96 (12)
C6—C1—C7—N1	5.15 (17)	C12—O3—C15—C16	−176.58 (11)
C2—C1—C7—N1	−177.43 (11)		

### Hydrogen-bond geometry (Å, º)

**Table:**

*D*—H···*A*	*D*—H	H···*A*	*D*···*A*	*D*—H···*A*
O2—H1···O1^i^	0.84	1.77	2.6106 (15)	174
N1—H2···O1*W*^ii^	0.85	2.10	2.9278 (16)	167
O1*W*—H3···O2^iii^	0.81	2.06	2.8683 (15)	177
O1*W*—H4···O2*W*	0.88	1.84	2.7159 (19)	169
O2*W*—H5···O1^iv^	0.87	2.14	2.8558 (18)	139
O2*W*—H5···N2^iv^	0.87	2.55	3.3363 (19)	151
O2*W*—H6···O3^v^	0.89	2.11	2.9791 (17)	165
C6—H6*A*···O1*W*^ii^	0.95	2.36	3.2942 (18)	169
C8—H8*A*···O1*W*^ii^	0.95	2.49	3.3222 (18)	146

Symmetry codes: (i) *x*, −*y*+3/2, *z*−1/2; (ii) −*x*+1, *y*+1/2, −*z*+1/2; (iii) *x*, −*y*+3/2, *z*+1/2; (iv) *x*−1, −*y*+3/2, *z*−1/2; (v) −*x*+1, −*y*+2, −*z*+1.

## References

[bb1] Allen, F. H., Kennard, O., Watson, D. G., Brammer, L., Orpen, A. G. & Taylor, R. (1987). *J. Chem. Soc. Perkin Trans. 2*, pp. S1–19.

[bb2] Bruker (2005). *APEX2*, *SAINT* and *SADABS* Bruker AXS Inc., Madison, Wisconsin, USA.

[bb3] Cosier, J. & Glazer, A. M. (1986). *J. Appl. Cryst.* **19**, 105–107.

[bb4] Fun, H.-K., Horkaew, J. & Chantrapromma, S. (2011). *Acta Cryst.* E**67**, o2644–o2645. 10.1107/S1600536811036579 PMC3201407 22064732

[bb5] Horkaew, J., Chantrapromma, S., Anantapong, T., Kanjana-Opas, A. & Fun, H.-K. (2012). *Acta Cryst.* E**68**, o1069–o1070. 10.1107/S160053681201032X PMC3344027 22589936

[bb6] Horkaew, J., Chantrapromma, S. & Fun, H.-K. (2011). *Acta Cryst.* E**67**, o2985. 10.1107/S1600536811041535 PMC3247387 22220005

[bb7] Loncle, C., Brunel, J. M., Vidal, N., Dherbomez, M. & Letourneux, Y. (2004). *Eur. J. Med. Chem.* **39**, 1067–1071. 10.1016/j.ejmech.2004.07.005 15571868

[bb8] Molyneux, P. (2004). *Songklanakarin J. Sci. Technol.* **26**, 211–219.

[bb9] Promdet, P., Horkaew, J., Chantrapromma, S. & Fun, H.-K. (2011). *Acta Cryst.* E**67**, o3224. 10.1107/S1600536811045740 PMC3238886 22199739

[bb10] Raj, K. K. V., Narayana, B., Ashalatha, B. V., Kumari, N. S. & Sarojini, B. K. (2007). *Eur. J. Med. Chem.* **42**, 425–429. 10.1016/j.ejmech.2006.09.010 17074422

[bb11] Sheldrick, G. M. (2008). *Acta Cryst.* A**64**, 112–122. 10.1107/S0108767307043930 18156677

[bb12] Spek, A. L. (2009). *Acta Cryst.* D**65**, 148–155. 10.1107/S090744490804362X PMC2631630 19171970

